# Built for bulk: posture, microanatomy and soft tissue shaped gigantism in Sauropoda

**DOI:** 10.1098/rsos.261186

**Published:** 2026-10-07

**Authors:** Julian C. G. Silva Junior, Gabriel Souza Ferreira, Agustin G. Martinelli, Thiago da S. Marinho, Felipe C. Montefeltro

**Affiliations:** ^1^ Laboratório de Paleontologia e Evolução de Ilha Solteira, UNESP, Ilha Solteira, São Paulo, Brazil; ^2^ Departamento de Biologia, Universidade de São Paulo, Ribeirão Preto, São Paulo, Brazil; ^3^ Senckenberg Centre for Human Evolution and Palaeoenvironment, Tübingen, Baden-Württemberg, Germany; ^4^ Sección Paleontología de Vertebrados, Museo Argentino de Ciencias Naturales Bernardino Rivadavia, Buenos Aires, Buenos Aires, Argentina; ^5^ Centro de Pesquisas Paleontológicas L. I. Price, Complexo Cultural e Científico Peirópolis, Pró-Reitoria de Extensão Universitária , Universidade Federal do Triângulo Mineiro, Uberaba, Brazil; ^6^ Departamento de Ciências Biológicas, Universidade Federal do Triângulo Mineiro, Uberaba, Brazil

**Keywords:** Sauropodomorpha, gigantism, finite element analyses, biomechanics

## Abstract

Sauropodomorph dinosaurs show how appendicular skeletal architecture accommodated extreme increases in body mass. Although sauropod gigantism has been linked to columnar limbs, wide-gauge posture, thick articular cartilage and tubular long-bone organization, the biomechanical interaction among these components is poorly understood. Here, we examine how femo ral shape, internal bone architecture and articular soft tissues influenced mechanical scaling across Sauropodomorpha. We generated three-dimensional femoral models spanning early sauropodomorphs, narrow-gauge diplodocoids, intermediate-gauge titanosauriforms, wide-gauge titanosaurs and an African elephant control. Finite element analyses compared theoretical solid models with taxon-specific native configurations, incorporating medullary cavities and articular cartilage caps. Mechanical performance was assessed using 95^th^-percentile von Mises stress, strain energy density and structural stiffness. Femoral performance did not scale uniformly with body mass. The two sampled narrow-gauge taxa, *Diplodocus* and *Amargasaurus*, defined steeper scaling trajectories for stress and strain energy density, whereas wide-gauge titanosaurs generally displayed flatter trajectories, comparatively lower stress and strain energy density, and elevated stiffness in several large-bodied forms. Targeted sensitivity analyses further showed that the mechanical effects of medullary architecture and cartilage depended on their interaction with taxon-specific femoral geometry. These findings suggest that sauropod gigantism was sustained by lineage-specific combinations of femoral geometry, internal architecture and articular soft tissues.

## 1. Introduction

Sauropodomorpha [1] represents one of the most successful radiations of Mesozoic herbivores, evolving from small cursorial taxa in the Late Triassic to the quadrupedal, columnar-limbed Sauropoda, the largest terrestrial animals ever recorded [2–4]. A key shift toward gigantism involved a reversal from the ancestral bipedal condition to a graviportal, quadrupedal locomotor system [5,6]. This postural transition may have functioned as a strategy in which lateral stresses were reduced through parasagittal alignment of the limb bones, accompanied by decreased elbow and knee flexion [7,8].

While the initial acquisition of a graviportal posture with columnar limbs allowed the support of large body masses, the evolution of extreme body sizes, particularly within Titanosauria, is associated with a second major postural shift: the transition from narrow-gauge postures, typical of diplodocids, to intermediate-gauge conditions in brachiosaurids, and ultimately to wide-gauge postures, in which the limbs were positioned farther from the body midline. Although this shift was first recognized from the ichnological record through differences in trackway width [9,10], subsequent osteological and morphometric studies have demonstrated that it was deeply rooted in appendicular anatomy, involving substantial modifications in the limbs, pelvic girdle and pectoral girdle [11–14].

The wide-gauge posture is consistent with improved mass distribution and potentially enhanced stability as these animals attained larger body sizes [15,16]. It has therefore been proposed that the combination of anatomical modifications and enhanced mass distribution may have been associated with the emergence of gigantic forms and the subsequent dominance of this fauna during the final stages of the Cretaceous [17,18].

Beyond the postural rearrangements associated with increased body size, the presence of specific soft tissues and microanatomical features probably formed part of a multi-level biomechanical system that allowed these lineages to sustain extreme body sizes beyond what would be predicted from bone geometry alone. In contrast to large terrestrial mammals such as elephants, in which relative articular cartilage thickness decreases with increasing body size [19], sauropods retained exceptionally thick articular cartilages that scaled positively or isometrically with body size, accounting for approximately 10%–12% of total bone length [20,21]. This thick and deformable cartilage probably played a significant role in compensating for the low osteological congruence, typical of archosaur joints [22]. By deforming under compressive loads, it may have increased effective contact area and redistributed stress across the articular surface and underlying subchondral bone. Such a configuration is consistent with a moderating effect on localized pressure peaks during locomotion, potentially reducing focal stress concentrations [23].

Complementing these soft-tissue adaptations, the internal microarchitecture of the appendicular skeleton followed a distinct evolutionary pattern that was also structurally relevant for weight support. Although large terrestrial mammals exhibit substantial morphological disparity, sauropods are frequently characterized by a tubular organization in which the cortical bone is distributed away from the neutral axis, surrounding a medullary cavity of variable extent [22,24]. Such a configuration may have influenced how skeletal robusticity scaled with body mass, potentially enabling titanosaurs to maintain high structural stiffness and functional safety margins as body size increased [19,25].

Despite extensive work on sauropod gigantism, how different structural modules of the appendicular skeleton interacted to accommodate increasing body mass remains poorly understood. In particular, the relative contributions of external femoral shape, articular cartilage and internal bone architecture have rarely been evaluated within a single biomechanical context.

Here, we combine three-dimensional modelling, finite element analysis (FEA) and comparative anatomy within a phylogenetic framework to examine the modular biomechanical evolution of the sauropodomorph femur. Specifically, we test how variation in femoral cross-sectional geometry, medullary cavity volume and articular cartilage affects mechanical performance under axial loading. By comparing taxa spanning the evolutionary tree, from early sauropodomorphs to giant Titanosauriformes, we evaluate whether these structural modules acted independently or in combination to shape the mechanical scaling of the appendicular skeleton during the evolution of sauropod gigantism.

## 2. Material and methods

### 2.1. Element selection

To test different evolutionary trends and mechanical responses, we selected femora from a range of sauropod lineages. We established the femur as a functional proxy for the appendicular skeleton, as this element represents one of the largest and best-sampled bones, is directly involved in postural adaptations [12,26,27] and supports the majority of the animal's weight.

### 2.2. Taxon sampling and digitization

The seven Neosauropoda specimens analysed by Silva Junior *et al.* [28] were also included in this study. Additionally, an African elephant (mammalian control) and *Plateosaurus* (outgroup comparison) were incorporated to broaden the comparative framework. These two specimens underwent digital restoration in Blender 5.0.1 to correct cracks and broken surfaces, ensuring their suitability for biomechanical testing [29]. Before volumetric mesh generation, all surface models were processed using voxel-based remeshing in Blender. The voxel size was adjusted individually for each specimen to produce a broadly uniform polygon size and a final surface resolution of approximately 300 000 faces per model. This procedure reduced the differences in mesh density and irregular polygon distribution among specimens, thereby limiting potential mesh-related artefacts in the finite element analyses. No subdivision algorithm was applied. Following remeshing, each model was visually compared with its original surface to verify that the overall femoral geometry and relevant anatomical features had been preserved. The resulting meshes were screened for errors, including non-manifold edges and intersecting faces, using Blender's ‘3D Print’ toolbox. All digitally restored models were uploaded to MorphoSource (supplementary material, appendix S1), to be accessible through their respective curators.

### 2.3. Functional modelling

To evaluate how shape, internal architecture and the presence of soft tissue influence the trade-off between mechanical safety and potential skeletal maintenance cost in the appendicular skeleton, we first modelled anatomical variations in an African elephant (*Loxodonta*), *Uberabatitan* and *Amargasaurus* as functional baselines. The elephant and *Uberabatitan* were selected because of their similar femoral lengths and estimated body masses, whereas *Amargasaurus* was included as a narrow-gauge comparator because its estimated body mass (7388 kg) differed by no more than approximately 11% from those of the elephant (6760 kg) and *Uberabatitan* (6653 kg). Because elevated strain energy density (SED) may be associated with increased remodelling demand [30,31], configurations that reduce stress but increase strain energy were interpreted as reflecting a trade-off between fracture risk and potential maintenance cost.

For the elephant, three models were generated to test the effect of medullary cavity volume: (i) solid configuration, consisting solely of compact bone; (ii) native mammalian configuration, incorporating the original large medullary cavity; and (iii) sauropod-like reduced medulla, incorporating a reduced medullary cavity, consistent with the proportions assumed for the sauropod models.

For *Uberabatitan* and *Amargasaurus*, six configurations were tested to characterize the mechanical roles of articular soft tissue and internal architecture: (i) solid configuration, consisting entirely of compact bone; (ii) cartilage-only configuration, consisting of compact bone with articular cartilage caps; (iii) medulla-only configuration, incorporating the native reduced medullary cavity assumed for the sauropod models but without cartilage; (iv) native sauropod configuration, combining the native reduced medullary cavity with articular cartilage; (v) expanded-medulla configuration without cartilage, incorporating an enlarged, mammal-like medullary cavity; and (vi) expanded-medulla configuration with cartilage, combining the enlarged medullary cavity with articular cartilage.

To facilitate direct comparison among the three taxa and remove differences attributable solely to load magnitude, the sensitivity-analysis outputs were normalized post hoc to a common reference load, corresponding to a body mass of 6760 kg. Under the linear elastic assumptions of the models, 95th-percentile von Mises stress values were multiplied by the ratio between the reference and original loads, whereas SED values were multiplied by the square of this ratio.

Following Aureliano *et al.* ([25] and references therein), trabecular bone space was set to approximately 30% for Sauropodomorpha and 50% for large mammals. This value was applied uniformly across sauropodomorph taxa to standardize internal architecture and isolate the effects of external geometry and posture-related morphology.

A simplified cartilage cap covering each epiphysis was modelled to represent 8% of the total femoral length (i.e. 4% per epiphysis). Although slightly lower than the 10%–12% estimated for Sauropodomorpha [21,32], this value was chosen to reduce mesh complexity and improve numerical stability, while maintaining a functionally comparable representation of epiphyseal cartilage across all taxa. To evaluate evolutionary trends across lineages, the solid and native models (figure 1) for all taxa were included in the broader statistical analyses.

**Figure 1.**
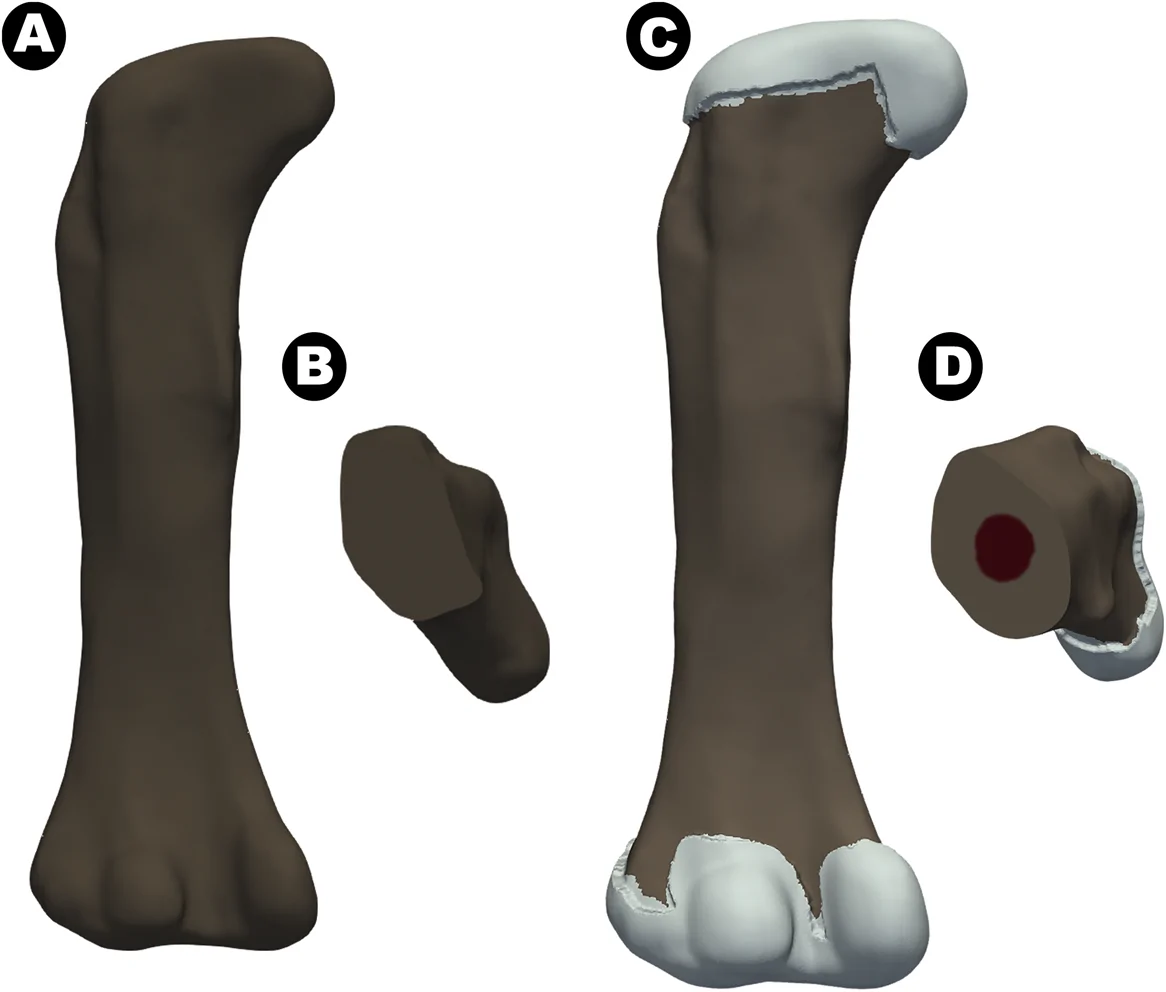
Modelled femoral configurations used in the finite element analyses. (A) Solid model in posterior view, consisting entirely of compact bone. (B) Transverse section of the solid model. (C) Native model in posterior view, incorporating medullary cavity and articular cartilage caps. (D) Transverse section of the native model, showing the medullary cavity and the articular cartilage at the epiphyses. Colour scheme: brown = cortical bone; red = trabecular bone; white = articular cartilage.

### 2.4. Finite element and biomechanical analyses

To simulate the structural response of the appendicular skeleton under standardized loading conditions and model configurations, we performed a series of FEA. The loading scenarios were pre-processed in HyperMesh 2021.2. Compressive loads were initially applied to representative seed nodes at the base of the femoral head (figure 2). Load magnitude followed Jannel *et al.* [23] and was calculated by multiplying the estimated body mass (kg) by gravitational acceleration (*g* = 9.81 m s^−^²) and dividing the resulting force by four. Although *Plateosaurus* is traditionally reconstructed as bipedal [33,34], we applied a standardized quadrupedal loading protocol across all taxa. This approach was adopted to normalize loading magnitude relative to body mass, ensuring that differences in output primarily reflect variation in femoral geometry and internal architecture rather than assumptions regarding locomotor posture. Because the applied load scales linearly with limb partitioning, alternative loading schemes, such as bipedal division of body mass, would modify absolute stress and displacement magnitudes, but are unlikely to alter the relative performance patterns observed among taxa under equivalent modelling conditions [35].

**Figure 2.**
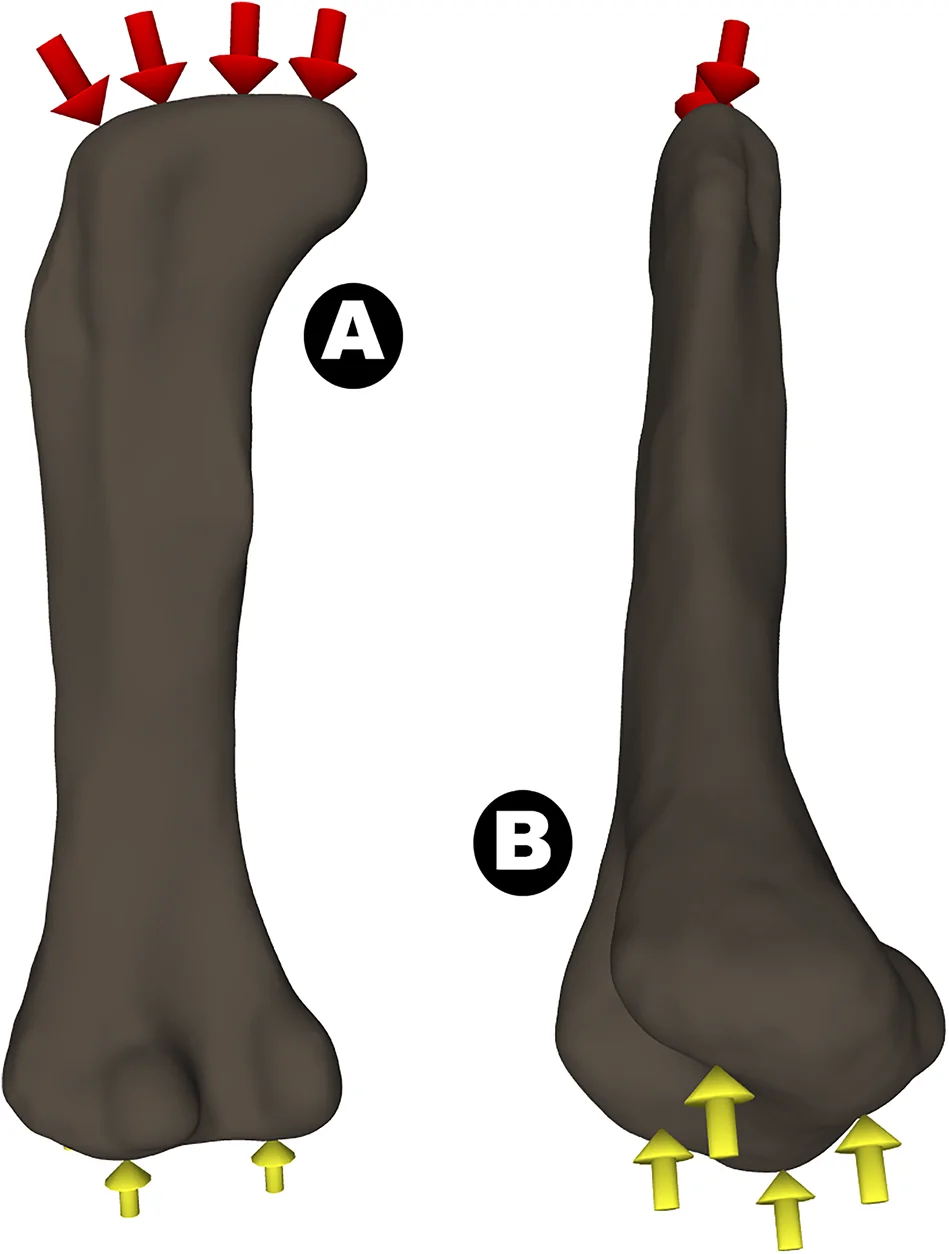
Loading and boundary conditions used in the finite element analyses. Red arrows indicate the compressive loads applied to the proximal femoral region, whereas yellow arrows indicate the translational constraints applied to the distal condyles. (A) Posterior view. (B) Lateral view.

Estimated body masses and the corresponding loads are provided in supplementary material, appendix S2. Boundary conditions were applied to the distal condyles: nodes on the distal articular surface were constrained in all translational degrees of freedom (*x*, *y* and *z*) (figure 2). The simulations were solved using Abaqus 2020, with outputs set to capture von Mises stress, nodal displacement and strain energy.

Fracture-risk-related mechanical demand was assessed using the 95th-percentile of von Mises stress (in MPa). Von Mises stress has been widely used in FEA as a comparative indicator of stress concentration and potential failure regions in skeletal structures [36–38]. The 95th-percentile was selected instead of absolute maximum values to reduce the influence of artificial stress singularities at load application nodes [39,40].

Structural stiffness (*k*) was calculated as the ratio between applied force (*F*) and maximum nodal displacement (*d*), following the force–displacement relationship commonly used to quantify global structural rigidity in biomechanical and finite element analyses [41]. SED was computed by dividing total strain energy (ALLSE) by bone volume and interpreted as a comparative indicator of mechanical stimulus and potential skeletal maintenance demand within adaptive bone-remodelling theory [30,31,42].

The material assumptions used in all simulations were standardized as follows. Material properties were assigned based on previous histological studies, indicating that dinosaur bone is structurally analogous to the fibrolamellar complex of fast-growing mammals [23,43]. Consequently, all cortical bone, including that of *Loxodonta* and *Plateosaurus*, was assigned a Young's modulus (*E*) of 10 000 MPa and a Poisson's ratio (*ν*) of 0.3. For the articular cartilage cap, values of *E* = 100 MPa and *ν* = 0.4 were applied, representing standard literature values for loaded cartilage [44]. For trabecular bone, properties were set to *E* = 500 MPa and *ν* = 0.3, corresponding to the established reference values [45,46].

To evaluate how these parameters scale with increasing body mass, comparative allometric analyses were performed using logarithmic regressions across lineages. These analyses allowed assessment of how fracture-risk-related stress, SED and structural stiffness varied along evolutionary size trajectories, providing a quantitative basis for comparing scaling responses among postural groups.

## 3. Results

### 3.1. Sensitivity to internal structure

The internal architecture of the femur influences load distribution and structural efficiency under the simulated conditions (figures 3 and 4). All taxa were modelled under both solid and native configurations, incorporating medullary cavities and articular cartilage caps, thereby enabling direct comparison of structural performance across different architectural conditions. An expanded sensitivity analysis was additionally conducted for *Uberabatitan*, representing a wide-gauge titanosaur, and *Amargasaurus*, representing a narrow-gauge sauropod.

**Figure 3.**
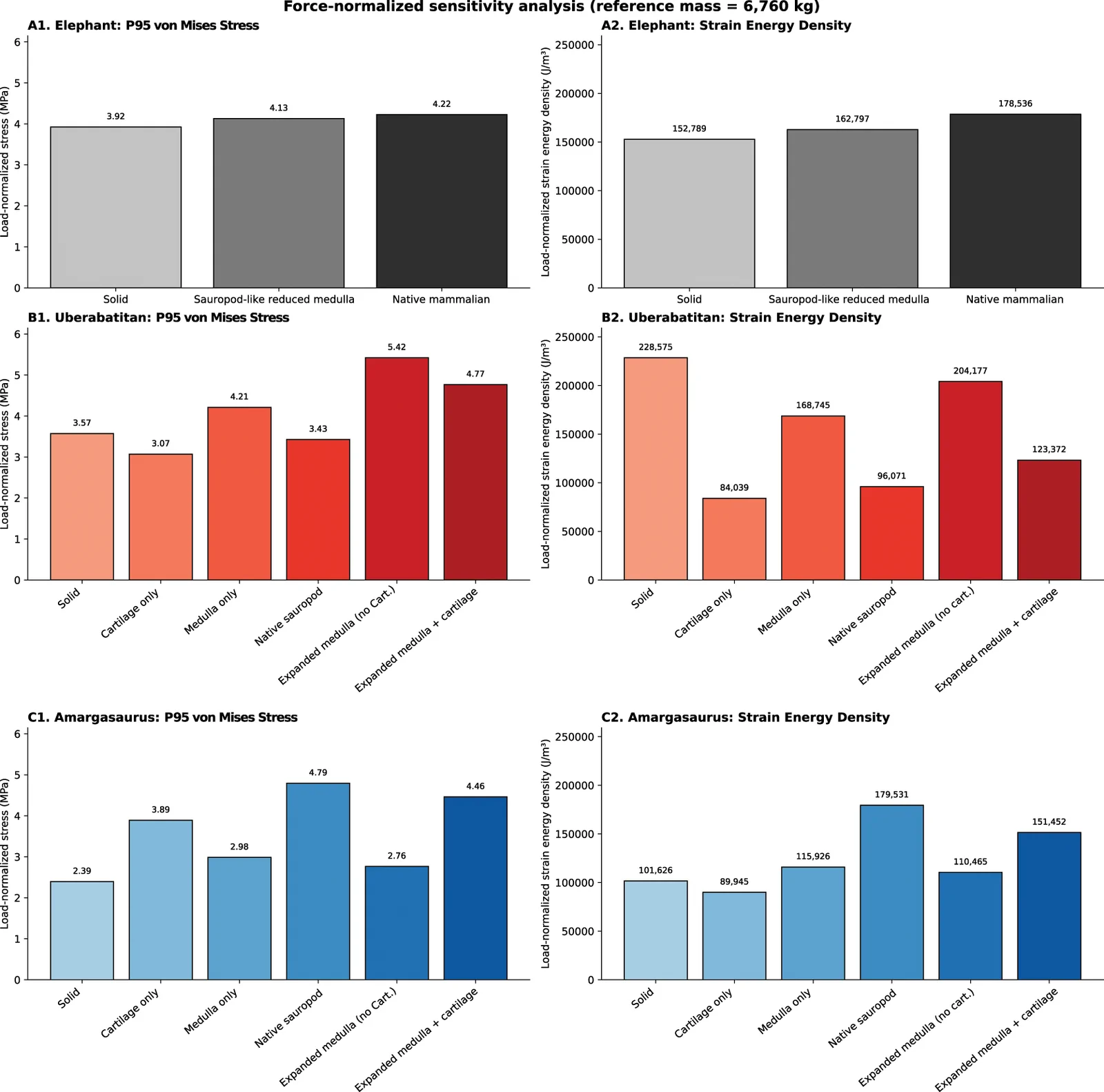
Load-normalized sensitivity analysis of internal bone architecture and articular cartilage on femoral mechanical performance. (A1,A2) Load-normalized 95th-percentile von Mises stress and strain energy density (SED) in the African elephant across the solid, sauropod-like reduced medulla, and native mammalian configurations. (B1,B2) The same metrics in *Uberabatitan* and (C1,C2) *Amargasaurus* across the solid, cartilage-only, medulla-only, native sauropod, expanded-medulla without cartilage and expanded-medulla with cartilage configurations. All results were normalized to a common reference load, corresponding to a body mass of 6760 kg. The contrasting responses of *Uberabatitan* and *Amargasaurus* indicate that the mechanical effects of medullary architecture and cartilage depend on their interaction with taxon-specific femoral geometry.

**Figure 4.**
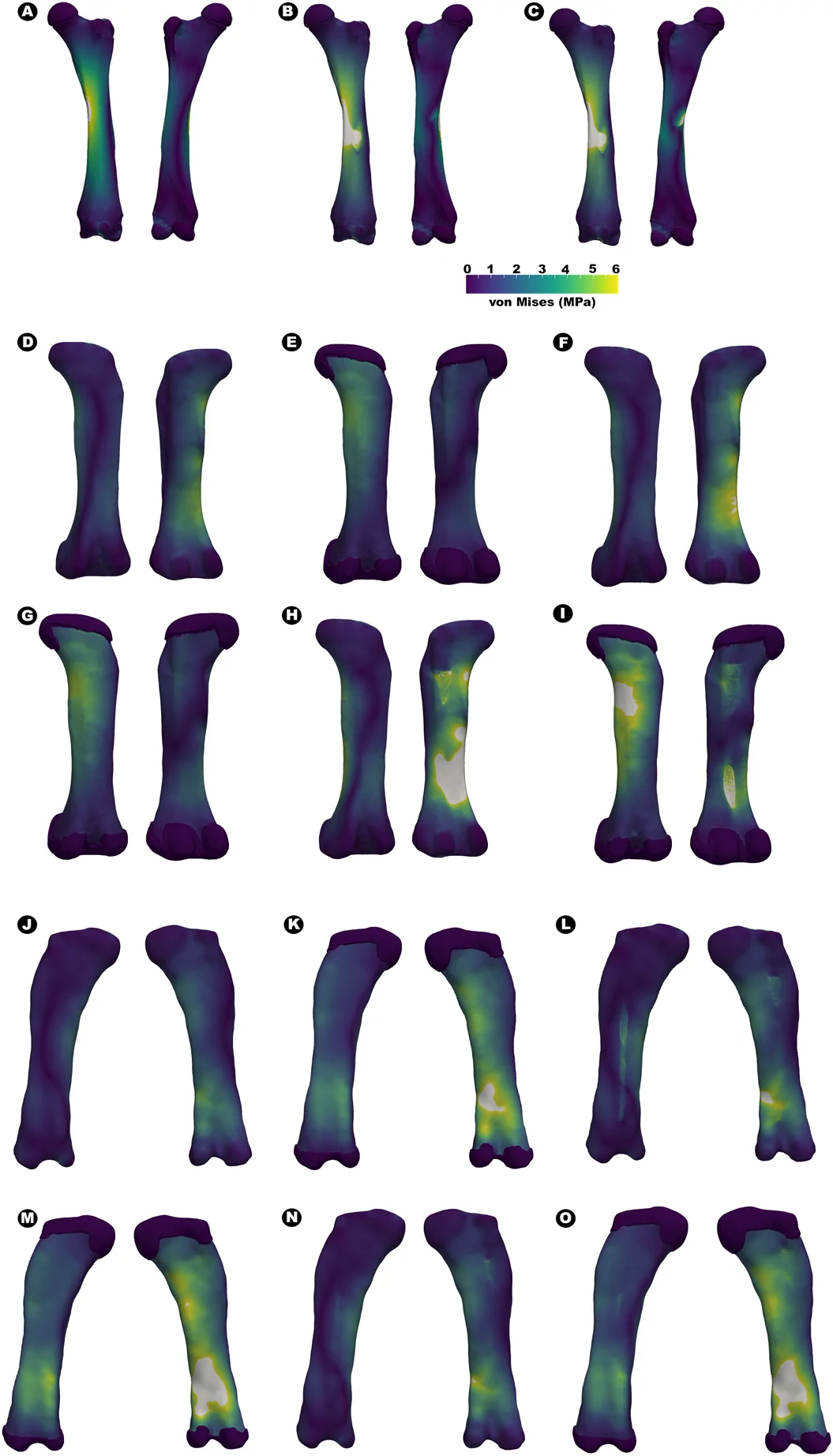
Distribution of von Mises stress in force-normalized sensitivity models of the African elephant, *Uberabatitan* and *Amargasaurus*. (A–C) Elephant models comprising the solid, sauropod-like reduced medulla and native mammalian configurations. (D–I) Six configurations of *Uberabatitan*: solid, cartilage only, medulla only, native sauropod, expanded medulla without cartilage and expanded medulla with cartilage. (J–O) The corresponding six configurations of *Amargasaurus*. All models were normalized to a common reference load, corresponding to a body mass of 6760 kg. The colour scale indicates von Mises stress in MPa, with white regions representing values exceeding the upper limit of the defined scale.

In the elephant control, structural responses remained broadly comparable across the three medullary configurations (figure 3A1). Load-normalized 95th-percentile von Mises stress increased slightly from 3.92 MPa in the solid model to 4.13 MPa in the sauropod-like reduced medulla configuration and 4.22 MPa in the native mammalian configuration. SED followed the same gradual pattern across the three configurations (figure 3A2). The limited magnitude of these differences indicates that alterations in medullary proportions produced only modest changes in the mechanical response of the elephant femur under the tested loading conditions.

In *Uberabatitan*, the effects of internal architecture and cartilage were more pronounced. The native configuration exhibited slightly lower stress than the solid model (figure 3B1) (3.43 versus 3.57 MPa) and substantially lower SED (figure 3B2). The cartilage-only configuration produced the lowest stress and SED values among the tested configurations, with a stress of 3.07 MPa. By contrast, the medulla-only configuration increased stress to 4.21 MPa, although its SED remained lower than that of the solid model.

Expanding the medullary cavity to mammal-like proportions produced the highest stress recorded for *Uberabatitan* (5.42 MPa) and was also associated with elevated SED. Adding articular cartilage to this expanded medullary configuration reduced stress to 4.77 MPa and also reduced SED. Thus, in *Uberabatitan*, cartilage consistently reduced SED and generally attenuated the mechanical consequences of the medullary cavity, although the native configuration was not the absolute lowest-stress model.

The response of *Amargasaurus* differed markedly from that of *Uberabatitan*. The solid model exhibited the lowest stress value (2.39 MPa), whereas the medulla-only and mammal-like expanded medulla configurations produced only moderate increases, reaching 2.98 and 2.76 MPa, respectively (figure 3C1). The cartilage-only configuration reduced SED slightly relative to the solid model, but increased stress to 3.89 MPa.

The native configuration of *Amargasaurus* exhibited the highest stress and SED values among its six models (figure 3C2), with stress reaching 4.79 MPa. Similarly, adding cartilage to the mammal-like expanded medullary configuration increased stress from 2.76 to 4.46 MPa and also increased SED. Therefore, unlike in *Uberabatitan*, the inclusion of cartilage in *Amargasaurus* did not consistently attenuate stress (figure 4E,K) and, when combined with a medullary cavity, increased both stress and SED relative to the corresponding configurations without cartilage.

Collectively, these results demonstrate that the mechanical effects of medullary architecture and articular cartilage were not uniform across the sampled sauropods. The native combination of external geometry, medullary architecture and cartilage reduced SED without increasing stress in *Uberabatitan* (figure 4G), whereas the equivalent combination increased both metrics in *Amargasaurus* (figure 4M). This contrast indicates that the contribution of internal architecture and soft tissues depended strongly on their interaction with taxon-specific femoral geometry.

### 3.2. The influence of limb posture on structural efficiency

Comparative analysis across lineages reveals non-uniform mechanical responses with increasing body mass (figure 5). Examination of SED (figure 5A) and fracture-risk-related mechanical demand (95th-percentile von Mises stress; figure 5B) indicates that stress magnitudes vary among taxa under the standardized axial loading regime.

**Figure 5.**
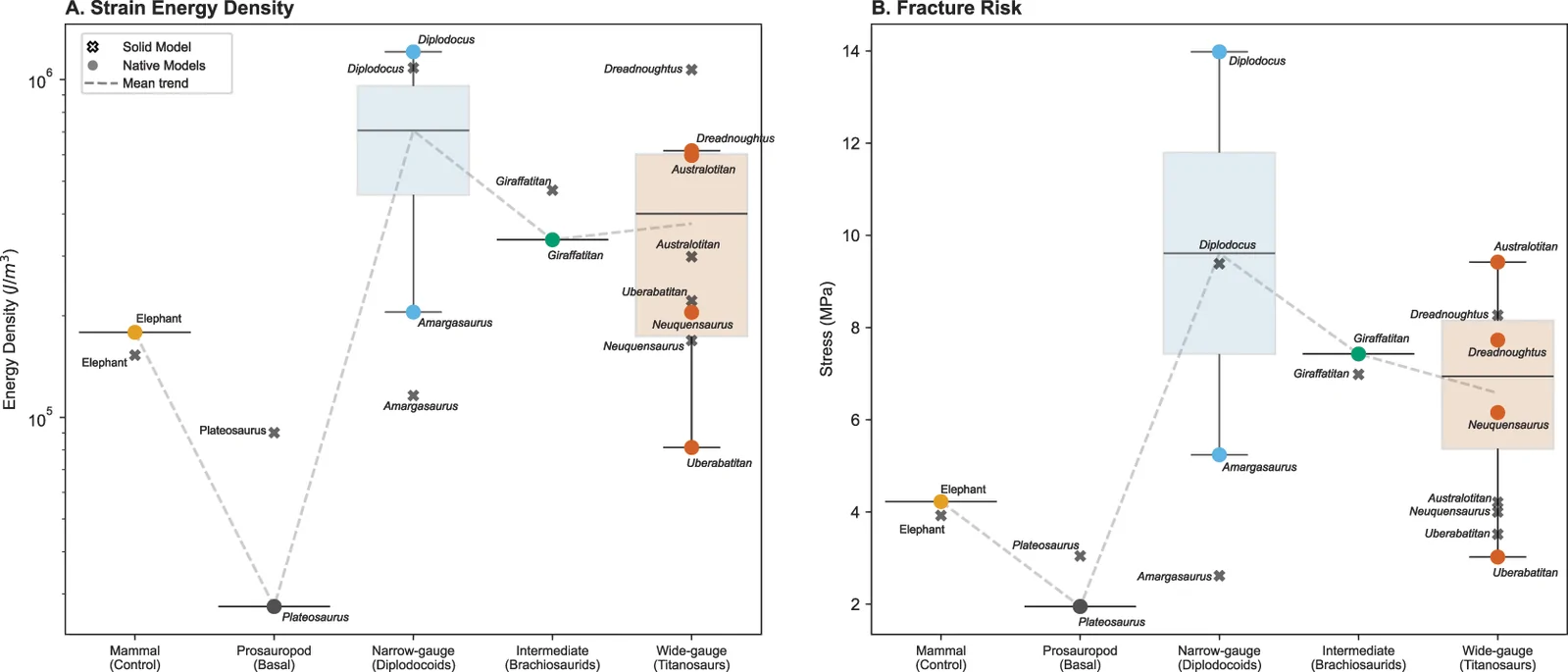
Evolutionary trends in biomechanical performance across Sauropodomorpha. (A) Distribution of strain energy density (SED; mechanical stimulus and potential skeletal maintenance demand). (B) Distribution of 95th-percentile von Mises stress, used as a proxy for fracture-risk-related mechanical demand. Box plots represent the interquartile range of native models, with the dashed grey line connecting group means. Solid models (X) and native models (O) are plotted individually. Within the present sample, the two narrow-gauge diplodocoids generally exhibit higher stress and SED than several wide-gauge titanosaurs, although substantial taxon-specific variation is present within both groups. Colour scheme: yellow = mammalian control; grey = early sauropodomorph; blue = narrow gauge; green = intermediate gauge; orange = wide gauge.

The two sauropods with inferred narrow-gauge postures, *Diplodocus* and *Amargasaurus*, generally plotted toward higher stress and SED values than several taxa of comparable body mass, although this pattern was most pronounced in *Diplodocus*. The analysed *Diplodocus* specimen, with an estimated body mass of approximately 25.1 metric tonnes (supplementary material, appendix S2), overlapped in mass with *Australotitan*, at approximately 24.2 metric tonnes, but exhibited higher 95th-percentile von Mises stress and SED under the same standardized mass-specific loading protocol.

By contrast, Titanosauriformes, such as *Giraffatitan* and *Dreadnoughtus*, maintain comparatively moderate stress and SED values across a similar or greater mass range (figure 5). However, *Australotitan* departs from this general pattern, exhibiting comparatively elevated stress and SED and lower structural stiffness relative to other sampled wide-gauge titanosaurs. In these taxa, the native configuration frequently performs similarly to, or below, the solid baseline model. Across narrow-gauge taxa, the native condition tends to exhibit higher stress and SED values than the corresponding solid configuration, whereas in wide-gauge taxa, native models frequently match or outperform the solid configuration in terms of stress magnitude and strain energy density.

The spatial distribution of von Mises stress in the native models further supports this pattern (figure 6). Narrow-gauge taxa show more extensive regions of elevated diaphyseal stress, whereas intermediate- and wide-gauge Titanosauriformes generally display more distributed stress fields along the femoral shaft. These contour plots reinforce the quantitative results shown in figure 5 by demonstrating that differences among postural groups are not restricted to scalar output values, but are also reflected in the spatial organization of stress across the femur.

**Figure 6.**
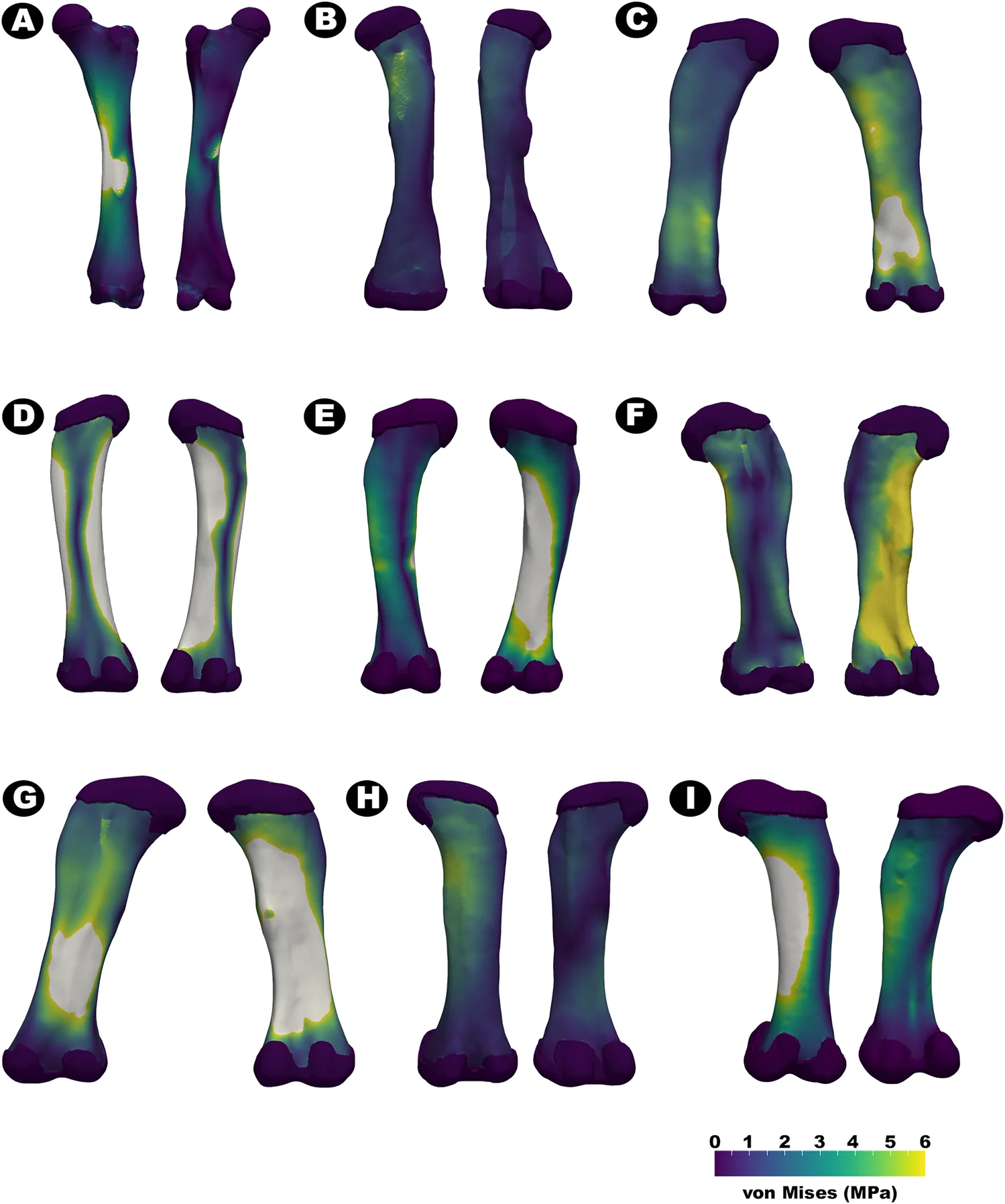
Von Mises stress distribution across native models. Anterior and posterior views of (A) elephant; (B) *Plateosaurus*; (C) *Amargasaurus*; (D) *Diplodocus*; (E) *Giraffatitan*; (F) *Dreadnoughtus*; (G) *Australotitan*; (H) *Uberabatitan* and (I) *Neuquensaurus*. The colour scale indicates von Mises stress values in MPa, with white regions representing values that exceed the upper limit of the defined scale.

### 3.3. Allometric scaling of biomechanical performance

Logarithmic regressions reveal divergent scaling trajectories among postural lineages as body mass increases (figure 7). In narrow-gauge diplodocoids, both SED (figure 7A) and 95th-percentile von Mises stress (figure 7B) exhibit steep positive scaling relationships with body mass (slopes = 1.45 and 0.80, respectively). As mass increases, the native models show greater departures from the theoretical solid baseline, displaying higher stress and SED values at larger body sizes.

**Figure 7.**
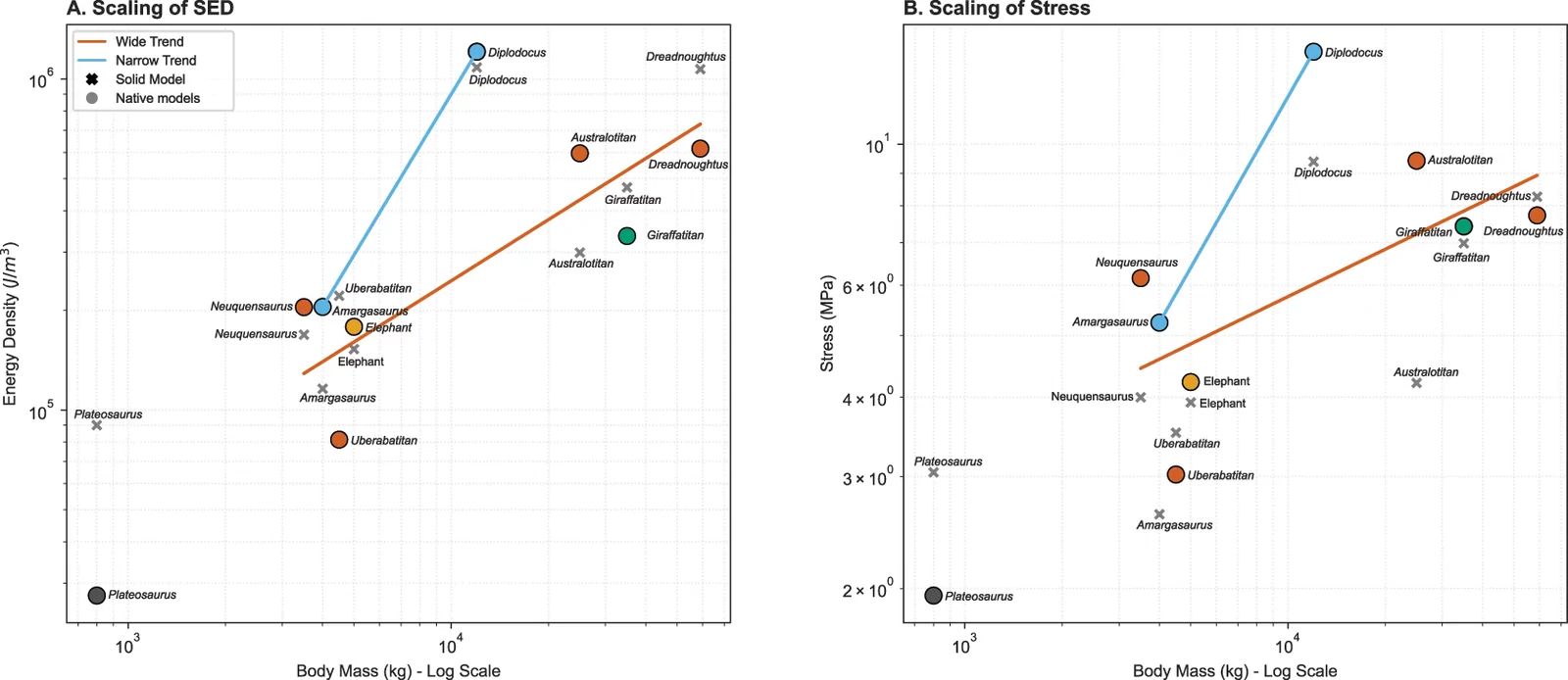
Allometric scaling of biomechanical performance across modelled specimens. (A) Scaling of strain energy density (SED; mechanical stimulus and potential skeletal maintenance demand) versus body mass. (B) Scaling of 95th-percentile von Mises stress, used as a proxy for fracture-risk-related mechanical demand, versus body mass. Logarithmic regressions highlight divergent trajectories between narrow-gauge diplodocoids (blue) and wide-gauge titanosaurs (orange). Narrow-gauge taxa show steeper positive scaling for both SED (slope = 1.45) and stress (slope = 0.80), whereas wide-gauge titanosaurs exhibit flatter trajectories (SED slope = 0.58; stress slope = 0.23). Colour scheme: yellow = mammal; grey = prosauropod; blue = narrow gauge; green = intermediate gauge; orange = wide gauge.

By contrast, the wide-gauge trajectory demonstrates comparatively flatter scaling slopes for both fracture-risk-related mechanical demand (slope = 0.23) and SED (slope = 0.58). Across the examined mass range, native models within this lineage show more moderate increases in stress and strain energy density, frequently plotting around the theoretical solid baseline. This pattern indicates reduced mechanical sensitivity to increasing body mass relative to the narrow-gauge condition.

### 3.4. Scaling of structural stiffness

Structural stiffness (*k = F/d*) shows positive scaling with body mass, but also reveals notable morphological variation (figure 8). *Uberabatitan* plots substantially above the general trend line, indicating high absolute stiffness relative to its mass. Similarly, *Dreadnoughtus* exhibits elevated stiffness values compared with taxa with intermediate-gauge and narrow-gauge postures.

**Figure 8.**
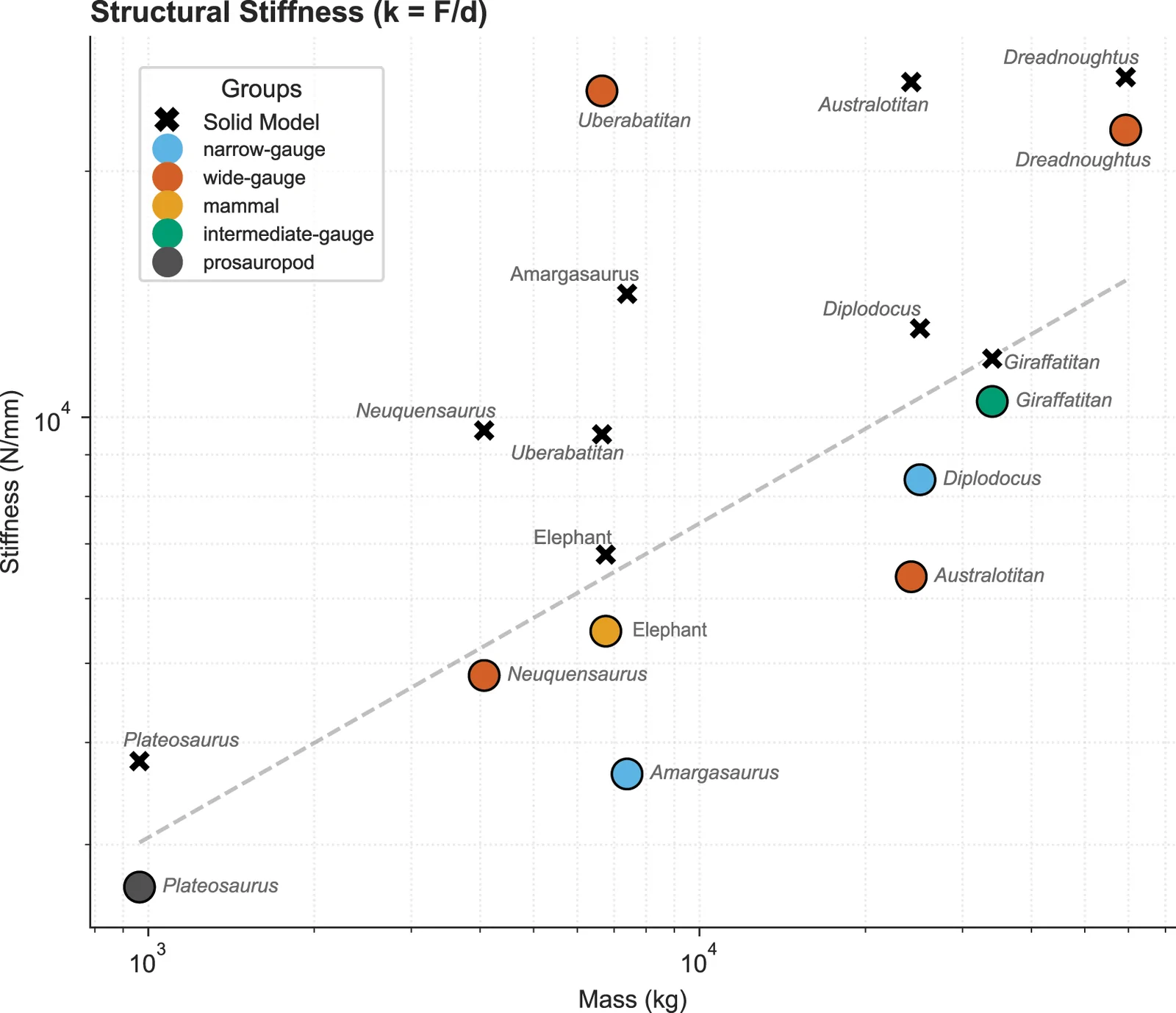
Scaling of global structural stiffness across body mass. Structural stiffness was calculated as the ratio between applied force and maximum nodal displacement (*k* = *F*/*d*). Solid models are represented by black crosses, and native models by coloured circles. Some wide-gauge titanosaurs, particularly *Uberabatitan* and *Dreadnoughtus*, exhibit high stiffness values relative to body mass under the applied loading regime, consistent with reduced deformation at large sizes.

Conversely, narrow-gauge forms such as *Amargasaurus* plot below the primary trend line, indicating comparatively greater deformation under equivalent normalized loads. These patterns are consistent with the stress and SED analyses, indicating that wide-gauge femoral architectures exhibit comparatively higher structural stiffness under standardized axial loading. However, this does not imply the absence of mechanical constraints; rather, the data indicate reduced deformation relative to body mass in certain titanosaurs when compared with narrow-gauge taxa.

## 4. Discussion

The results presented herein indicate that femoral structural performance across Sauropodomorpha does not follow a uniform mechanical trajectory with increasing body mass. Instead, distinct postural lineages exhibit divergent scaling patterns in fracture-risk-related mechanical demand, strain energy density and structural stiffness under standardized axial loading. These differences are consistently associated with variation in femoral cross-sectional geometry, internal architecture and the inclusion of articular soft tissues under standardized modelling conditions. Collectively, the observed patterns are compatible with the interpretation that shifts in appendicular architecture may have modified load transmission dynamics during the evolution of sauropod gigantism. The following sections explore how posture, articular soft tissues and internal bone architecture contribute to these biomechanical trends and consider their broader evolutionary context.

### 4.1. Limb posture and shifts in mechanical regime

The results demonstrate that femoral structural performance does not scale uniformly across Sauropodomorpha. Instead, narrow-gauge and wide-gauge lineages follow distinct scaling trajectories under standardized axial loading. Narrow-gauge taxa exhibit steeper positive slopes of fracture risk and strain energy density relative to body mass, whereas wide-gauge titanosaurs show comparatively flatter trajectories and higher structural stiffness at large sizes.

These patterns suggest that limb architecture influenced how increasing body mass was mechanically accommodated within different sauropod clades. Early quadrupedalization in sauropodomorphs involved increased limb columnarity and parasagittal alignment [47–49], probably reducing bending moments relative to more crouched postures [50,51]. However, once quadrupedality was established, differences in limb posture appear to have changed how the appendicular skeleton responded mechanically to increasing body mass.

Narrow-gauge neosauropods are characterized by medially oriented limbs and relatively circular femoral cross-sections [7,12]. In the present models, these taxa exhibit increasing diaphyseal stress relative to body mass (figures 5 and 7), indicating that axial load magnitudes scale more steeply under this architectural configuration. By contrast, wide-gauge titanosaurs evolved mediolateral femoral expansion and more elliptical cross-sections [11,12,26], which increase the second moment of area without proportionally increasing bone mass. The flatter scaling slopes observed in these taxa (figure 7) are consistent with theoretical expectations that redistribution of cortical material enhances resistance to axial and bending stresses at larger body sizes [52,53].

Importantly, wide-gauge morphology does not correlate linearly with body size and has been interpreted as an exaptation rather than a direct driver of gigantism [12,54]. The present results are consistent with this interpretation: rather than initiating gigantism, wide-gauge architecture appears to have modified how structural demands scaled once large body sizes were attained. In this sense, wide-gauge posture did not remove mechanical constraints, but changed how increasing body mass was accommodated by the femur, reducing the scaling sensitivity of stress and strain energy in large-bodied titanosaurs.

However, mechanical performance was not uniform among the sampled wide-gauge titanosaurs. *Australotitan*, in particular, exhibited comparatively elevated stress and SED, and lower structural stiffness than several other titanosaurs included in the analysis. This variation indicates that wide-gauge posture alone did not determine femoral mechanical efficiency. Instead, taxon-specific femoral geometry, cortical distribution and medullary proportions probably interacted with posture to influence structural response. Accordingly, the flatter scaling trajectory observed for wide-gauge taxa should be interpreted as a general pattern within the present sample rather than as a mechanically uniform condition shared by all titanosaurs.

Rather than exhibiting the mediolateral femoral expansion observed in wide-gauge titanosaurs, diplodocoids attained large body sizes within a body plan characterized by pronounced axial elongation [55,56]. Axial proportions influence overall mass distribution and centre-of-mass position [15,57], and narrow-gauge neosauropods have been reconstructed with a posterior mass bias [7]. This suggests that diplodocoids followed a distinct biomechanical route to large body size, in which axial elongation may have influenced mass distribution and limb loading without the same femoral reorganization observed in wide-gauge titanosaurs.

### 4.2. Articular cartilage and load management

The finite element models indicate that the inclusion of substantial, unossified cartilaginous caps on sauropod epiphyses [21,58,59] significantly influences stress distribution under axial loading. However, the expanded sensitivity analysis demonstrates that the direction and magnitude of this effect were not uniform between the sampled sauropods.

In *Uberabatitan*, the addition of articular cartilage generally improved mechanical performance. Both the cartilage-only and native configurations reduced SED relative to the solid model, while cartilage also attenuated the increase in stress associated with an expanded medullary cavity (figure 3B1,B2). These results indicate that, within the *Uberabatitan* geometry, cartilage deformability contributed to load redistribution and mitigated the mechanical consequences of the medullary cavity.

The response of *Amargasaurus* differed. Although the cartilage-only configuration slightly reduced SED relative to the solid model, it increased 95th-percentile von Mises stress, and the inclusion of cartilage in models containing a medullary cavity increased both stress and SED (figure 3C1,C2). Thus, cartilage did not produce a universally beneficial response, but instead altered the balance between localized stress and global strain energy according to the underlying femoral geometry and medullary proportions.

Rather than functioning as a purely dissipative shock absorber, thick cartilage increases effective contact area and redistributes compressive loads across the articular surface [26,58]. FEA of sauropod pedes further demonstrate that compliant soft tissue structures substantially reduce peak stress transmission to osseous tissue under weight-bearing conditions [23,60]. Although those studies focused on autopodial mechanics, the present results indicate that the consequences of soft-tissue deformation at the femoral epiphysis depend on the structural context in which that deformation occurs. Redistribution may reduce mechanical demand in some geometries, as observed in *Uberabatitan*, but may increase localized stress in others, as observed in *Amargasaurus*.

In large mammals, cartilage thickness scales positively with body size and contributes to joint-level force transmission [61,62]. Sauropods retained proportionally substantial unossified epiphyseal cartilage, suggesting a distinct joint construction strategy in which the deformable nature of articular cartilage influenced how axial loads were transmitted to the diaphysis. Our results therefore support a role for articular cartilage in joint-level load redistribution, while showing that its mechanical contribution cannot be evaluated independently of external femoral shape and internal architecture. The more advantageous response observed in *Uberabatitan* suggests that the integration of wide-gauge femoral geometry, native medullary proportions and articular cartilage produced a mechanically favourable combination in the sampled titanosaur, rather than cartilage acting as an independently beneficial feat ure across Sauropodomorpha.

### 4.3. Microanatomy and the tubular strategy

Tubular optimization of long bones enhances structural efficiency by maximizing the second moment of area relative to bone mass [52,53]. However, sauropod limb bones exhibit considerable internal variability in cortical thickness and medullary cavity proportions, indicating that extreme body size was not associated with a single microanatomical configuration [22,25]. Histological studies further demonstrate that growth dynamics within Sauropodomorpha were themselves variable, with both uninterrupted rapid growth and cyclical strategies occurring across basal and derived taxa [63,64]. The present analyses do not aim to reconstruct lineage-specific histological patterns, but instead evaluate how variation in internal proportions influences structural performance under standardized loading.

The sensitivity tests indicate that internal geometry substantially affects stress distribution in multi-ton femora. In the elephant control, variation in medullary proportion produced relatively modest changes in stress and SED. The sauropod models, however, exhibited strongly geometry-dependent responses. In *Uberabatitan*, expanding the medullary cavity increased mechanical demand, whereas the native configuration substantially reduced SED relative to the solid model without increasing stress. In *Amargasaurus*, configurations containing a medullary cavity without cartilage produced comparatively moderate changes, whereas the native and cartilage-bearing medullary configurations increased both stress and SED. These contrasting responses indicate that the mechanical effects of internal architecture cannot be interpreted independently of external femoral geometry.

Importantly, later titanosaurs are characterized by extensive secondary remodelling, as documented in several taxa (e.g. [65,66]). Such remodelling indicates that cortical architecture in these forms was dynamically modified throughout ontogeny rather than remaining structurally static. The performance of the native *Uberabatitan* model therefore did not result from simple cortical thickening or shaft solidification. Instead, its assigned internal proportions allowed the femur to maintain stress values comparable to those of the solid configuration while substantially reducing SED. The contrasting response observed in *Amargasaurus* demonstrates that similar structural modifications do not necessarily produce equivalent mechanical outcomes across different femoral morphologies. Thus, extreme body size was accommodated through lineage-specific arrangements of cortical and medullary tissues rather than through a single optimal microanatomical configuration.

### 4.4. Study limitations and caveats

Limitations should be considered when interpreting the present results. First, the standardized vertically directed load isolates the effects of femoral geometry and internal architecture, but does not reproduce the bending, shear and torsional stresses generated by oblique ground-reaction forces, muscle activity and joint moments during locomotion. Dynamic and non-axial loading could alter both stress magnitude and distribution and may affect the relative performance of narrow- and wide-gauge femora. However, the direction of these effects cannot be predicted confidently without taxon-specific estimates of posture, gait, muscle moment arms and joint loading. Second, the narrow-gauge scaling trajectories are based on only two taxa, *Diplodocus* and *Amargasaurus*. Their steep relationships for SED and 95th-percentile von Mises stress should therefore be regarded as descriptive patterns within the sampled specimens rather than robust estimates for narrow-gauge sauropods as a whole. The wide-gauge sample is also limited and exhibits substantial taxon-specific variation, as illustrated by the comparatively elevated stress and SED and lower stiffness observed in *Australotitan*. Such variation cannot presently be attributed confidently to a single anatomical or modelling factor. Finally, preservation, reconstruction and geometric restoration may influence the responses of individual models. These caveats do not negate the broader patterns identified here, but they indicate that the results should be interpreted as comparative evidence under standardized axial loading rather than as complete reconstructions of lineage-wide locomotor performance.

## 5. Conclusion

The results demonstrate that sauropod gigantism was not sustained by a single anatomical innovation, but by different combinations of structural traits across lineages. Finite element analyses indicate that limb posture, femoral geometry, internal bone architecture and articular soft tissues jointly influenced how mechanical demands scaled with increasing body mass. The expanded sensitivity analysis further demonstrates that the effects of internal architecture and cartilage were geometry-dependent rather than uniformly beneficial. Their native combination reduced SED without increasing stress in *Uberabatitan*, whereas the corresponding configuration increased both metrics in *Amargasaurus*. However, these factors did not contribute equally across all taxa: narrow-gauge diplodocoids attained large body sizes within an axially elongated body plan associated with higher femoral stress and SED scaling, whereas wide-gauge titanosaurs generally showed reduced mechanical sensitivity through appendicular reorganization.

This pattern also reinforces the interpretation that wide-gauge architecture was not simply an adaptation to extreme body size after gigantism had already evolved. Previous morphometric studies have shown that wide-gauge features and hindlimb shape variation in Titanosauriformes do not correlate linearly with body size and may represent an exaptation for gigantism [12,54]. Our biomechanical results add functional support to this view by showing that wide-gauge titanosaurs shared flatter mechanical scaling trajectories, which may have expanded the range of body sizes attainable without proportional increases in femoral mechanical demand.

Together, these findings suggest that sauropod gigantism involved a shift in mechanical organization of the limb system. Increasing body mass was accommodated through lineage-specific changes in posture, femoral geometry, internal architecture and articular tissues, allowing the appendicular skeleton to maintain functional competence at body sizes far exceeding those of other terrestrial vertebrates.

## Supplementary Material



## Data Availability

The electronic supplementary material submitted with this article provides supporting methodo logical and descriptive information, including institutional abbreviations, specimen information, body mass estimates, loading conditions, details of cartilage and trabecular-bone/medullary region assignment, supplementary finite element results and supplementary material, figures S1–S4 [67]. Supplementary material, table S1 lists the modelled specimens, original sources, and MorphoSource accession links, with access subject to the availability conditions and permissions established by their respective curators and repositories. Supplementary material, table S2 provides the estimated body masses and the corresponding load magnitudes applied in the simulations. The data and code required to reproduce the quantitative analyses are deposited separately in Zenodo. These include finite element output files, nodal displacement and von Mises stress data, strain-energy data, supporting tables and the custom Python script developed to assign cartilage and trabecular-bone/medullary regions to the finite element models. These files are available for download from Zenodo [68].

## Declaration of AI use

GitHub Copilot was used to assist in the development and refinement of the Python script used to assign cartilage and trabecular-bone/medullary regions to the finite element models. All code was reviewed, tested and validated by the authors before use.
