# Supplementary material for: Built for bulk: posture, microanatomy and soft tissue shaped gigantism in Sauropoda
Source: R Soc Open Sci. 2026 Oct 7;13(10):261186. doi: 10.1098/rsos.261186 (PMC13641891; doi:10.1098/rsos.261186)
Supplement: Supplementary file 1 [file rsos.261186.zip › rsos.261186/rsos261186_si_001.docx]

Supplementary for BUILT FOR BULK: POSTURE, MICROANATOMY, AND SOFT TISSUE SHAPED GIGANTISM IN SAUROPODA

This supplementary document provides additional information on the finite element analyses performed on the selected femora, including details of the loading conditions and boundary constraints used in the simulations. Additional figures illustrating stress and displacement results for the different model configurations are also included.

*Institutional abbreviations: CPPLIP*, Centro de Pesquisas Paleontológicas Llewellyn Ivor Price, Universidade Federal do Triângulo Mineiro, Uberaba, Brazil; *EMF*, Eromanga Natural History Museum Fossil, Eromanga, Australia; *MACN-Pv*, Museo Argentino de Ciencias Naturales ‘Bernardino Rivadavia’, Coleccíon Nacional Paleontologia de vertebrados, Buenos Aires, Argentina; *MB.R*., Museum für Naturkunde Berlin, Berlin, Germany; *MLP*, Museo de La Plata, La Plata, Argentina; *MNHN*, Muséum national d'Histoire naturelle, Paris, France; *MPM*, Museo Padre Molina, Río Gallegos, Argentina; *NHMW*, Naturhistorisches Museum Wien, Vienna, Austria; *SMNS*, Staatliches Museum für Naturkunde Stuttgart, Stuttgart, Germany.

*Specimens used*

The Neosauropoda specimens analyzed in Silva Junior et al. (2025) were also included in the present study. Additionally, an elephant (*Loxodonta africana*) was used as an example of a large columnar-limbed quadrupedal mammal; and *Plateosaurus* was included as a representative early sauropodomorph, allowing a broader comparative framework.

**Table 1**. Modelled specimens used in this study.

| Species | Specimen | Original source | Morphosource doi |
| --- | --- | --- | --- |
| *Loxodonta africana* | MNHN-ZM-AC-1907.49 | https://www.morphosource.org/media/000632481 | <https://doi.org/10.17602/M2/M632481> |
| *Plateosaurus trossingensis* | unnumbered | https://sketchfab.com/3d-models/plateosaurus-skeleton-80683eb568cc46caac0649e395fa5a1b | <https://doi.org/10.17602/M2/M864356> |
| *Amargasaurus cazaui* | MACN-Pv N-15 | Silva Junior et al., 2025 | https://doi.org/10.17602/M2/M672959 |
| *Diplodocus sp.* | MACN-Pv 18 814 | Silva Junior et al., 2025 | <https://doi.org/10.17602/M2/M672964> |
| *Giraffatitan brancai* | MB.R.5016 | https://www.morphosource.org/concern/media/000664954 | <https://doi.org/10.17602/M2/M664954> |
| *Dreadnoughtus schrani* | MPM-PV 1156 | Lacovara et al., 2014 | <https://doi.org/10.17602/M2/M672997> |
| *Australotitan cooperensis* | EMF105 | Hocknull et al., 2021 | https://doi.org/10.17602/M2/M673555 |
| *Uberabatitan ribeiroi* | CPPLIP-1238 | Silva Junior et al., 2025 | <https://doi.org/10.17602/M2/M672939> |
| *Neuquensaurus australis* | MLP 1480 | Silva Junior et al., 2025 | <https://doi.org/10.17602/M2/M672991> |

*Body mass estimation*

The body mass of the specimens was estimated using the quadrupedal allometric scaling equation proposed by Campione and Evans (2012, 2020). This predictive method is based on the sum of the minimum diaphyseal circumferences of the proximal long bones, expressed as:

log_10_ *M =* 2.749 *x* log_10_ (*FC + HC*) – 1.104

where *M* represents the estimated body mass in grams, *FC* is the minimum femoral diaphyseal circumference (in mm), and *HC* is the minimum humeral diaphyseal circumference (in mm). The resulting mass was converted to kilograms and multiplied by the acceleration due to gravity to derive the weight force (in Newtons). This value was then divided to determine the static compressive loads applied to the three-dimensional models during the finite element analyses (FEA).

The load magnitude followed Jannel et al. (2022) and was calculated by multiplying the estimated body mass (kg) by gravitational acceleration (g = 9.81 m/s^2^) and dividing the resulting force by four. The estimated body masses and load magnitudes are presented in Table 2.

**Table 2**. Estimated body masses and load magnitudes used in this study.

| Species | Specimen | Estimated body mass (kg) | ¼ body weight load (N) |
| --- | --- | --- | --- |
| *Loxodonta africana* | MNHN-ZM-AC-1907.49 | 6760 | 16600 |
| *Plateosaurus trossingensis* | Unnumbered | 964^a^ | 2900 |
| *Amargasaurus cazaui* | MACN-Pv N-15 | 7388 | 18100 |
| *Diplodocus sp.* | MACN-Pv 18 814 | 25114 | 61600 |
| *Giraffatitan brancai* | MB.R.5016 | 33961 | 85000 |
| *Dreadnoughtus schrani* | MPM-PV 1156 | 59300 | 145400 |
| *Australotitan cooperensis* | EMF105 | 24213 | 59400 |
| *Uberabatitan ribeiroi* | CPPLIP-1238 | 6653 | 16300 |
| *Neuquensaurus australis* | MLP 1480 | 4067 | 9980 |

^a^For the *Plateosaurus* body mass estimation, the 3D models used here were scaled to the same proportions of SMNS 13200 (Schaeffer, 2024).

*Cartilage and medullary cavity*

To generate the native femoral configurations, the articular extremities of each femur were first mapped in Blender. Three-dimensional coordinate points were manually placed and exported from the epiphyseal regions corresponding to the inferred cartilaginous caps. These coordinates were used as spatial references for assigning the cartilage domain in the finite element mesh. Tissue assignment was performed using a custom Python script developed by Silva Junior for this study. The script reads the mapped coordinates exported from Blender and compares them with the centroid of each finite element in the Abaqus input file. A KD-tree nearest-neighbour search is then used to identify the elements closest to the mapped articular surfaces. The number of elements assigned to the cartilage region is defined as a user-specified percentage of the total mesh, allowing the cartilaginous cap to be applied consistently across models.

The internal trabecular-bone/medullary region was delimited geometrically from the finite element mesh. First, the script identifies external surface elements by detecting triangular element faces that are not shared with neighbouring elements. These surface elements are excluded from the internal assignment in order to preserve the cortical shell. The script then determines the longest anatomical axis of the model and allows the user to define proximal and distal limits for the internal region along this axis. Within this longitudinal interval, element centroids are projected onto the two remaining radial axes, and the most central elements are selected according to a user-defined percentage. These selected internal elements are assigned trabecular-bone material properties, whereas the remaining elements are assigned cortical-bone properties.

The script writes a new Abaqus input file in which previous material and section definitions are removed and replaced by new material assignments for cartilage, trabecular bone, and cortical bone. The elastic properties of each tissue, including Young’s modulus and Poisson’s ratio, can be edited through the graphical interface before generating the final input file.

*Loads and constraints*

The strategy for applying loads and kinematic constraints varied according to model composition. For models composed exclusively of bone tissue (solid models), compressive forces were defined at single nodes located on the proximal articular surface. However, to avoid numerical artifacts associated with highly localized constraints, boundary conditions were spatially distributed in both solid and cartilage-bearing models.

In models incorporating articular cartilage, the application of point loads would generate artifactual stress singularities, producing excessive and highly localized mesh deformation. To reduce these numerical effects, a nodal expansion approach was employed. In this procedure, compressive loads and boundary constraints were propagated from initial “seed” nodes to adjacent nodes within a predefined radius, allowing more distributed mesh deformation and stress dissipation across the articular surface while maintaining comparable boundary conditions among all model configurations.

*Supplementary Results*

To visually isolate the mechanical effects of macroscopic bone geometry from internal architecture, Von Mises stress and nodal displacement were mapped across theoretical solid models (Figures S1 and S2) and native models (Figure S3). In the fully solid configurations, where stress distribution is primarily dictated by external morphology, narrow-gauge taxa (e.g., *Diplodocus*, *Amargasaurus*) exhibit broad regions of elevated diaphyseal stress, consistent with higher bending-related demands under axial loading. Conversely, wide-gauge titanosaurs (e.g., *Dreadnoughtus*, *Uberabatitan*) show more spatially restricted high-stress regions, suggesting that mediolateral diaphyseal expansion contributes to more distributed load transmission under the tested loading conditions.

Furthermore, comparison of nodal displacement between solid and native models reveals that the inclusion of medullary cavities and articular cartilage generally increases proximal displacement, as expected from the presence of deformable tissues and hollow diaphyseal architecture. However, the global deformation pattern remains broadly similar across configurations. The cartilaginous cap appears to accommodate part of the compressive displacement at the epiphyseal region before loads are transmitted to the osseous diaphysis.

Kernel density distributions of nodal Von Mises stress and displacement values were used to compare the mechanical response of solid and native femoral models under identical loading conditions (Fig. S4). For each simulation, all nodal values exported from the finite element analysis were included, and extreme values were excluded using percentile-based thresholds in order to minimize the influence of numerical singularities associated with point loading.

With the exception of the Elephant model, which shows relatively small differences between configurations, Sauropodomorpha exhibit clear shifts in stress distribution between solid and native models. Solid configurations tend to produce more localized stress concentrations, whereas the inclusion of medullary cavities and articular cartilage results in broader stress distributions and lower peak values across the shaft. In contrast, displacement distributions remain broadly similar between configurations, with the highest values concentrated near the proximal articular region where axial loads were applied. These patterns indicate that internal architecture primarily affects the distribution and magnitude of stress rather than the overall deformation pattern, consistent with the sensitivity results presented in Fig. 3 (main text).

**Figure S1.** Von Mises stress distribution in theoretical solid models. Contour plots showing stress gradients across fully compact femoral geometries, used to isolate the biomechanical influence of external morphology. Taxa: (A) Elephant, (B) *Plateosaurus*, (C) *Amargasaurus*, (D) *Diplodocus*, (E) *Giraffatitan*, (F) *Dreadnoughtus*, (G) *Australotitan*, (H) *Uberabatitan*, and (I) *Neuquensaurus*. Narrow-gauge forms (C, D) show more extensive diaphyseal stress concentrations, whereas several wide-gauge titanosaurs display more spatially restricted high-stress regions. The color scale indicates Von Mises stress values in MPa, with white regions representing values that exceed the upper limit of the defined scale.

**Figure S2.** Nodal displacement mapping in theoretical solid models. Contour plots showing structural deformation under standardized mass-specific axial loading. Displacement values are lowest near the constrained distal condyles and increase toward the proximal loading region, reflecting the imposed axial loading and distal boundary conditions. Taxa: (A) Elephant, (B) *Plateosaurus*, (C) *Amargasaurus*, (D) *Diplodocus*, (E) *Giraffatitan*, (F) *Dreadnoughtus*, (G) *Australotitan*, (H) *Uberabatitan*, and (I) *Neuquensaurus*.. The color scale indicates displacement magnitude in mm, with white regions representing values that exceed the upper limit of the defined scale.

**Figure S3.** Contour plots showing displacement magnitude in models incorporating native medullary cavity proportions and articular cartilage caps. Compared with theoretical solid models, native configurations generally show increased proximal displacement associated with the presence of deformable articular tissues and hollow diaphyseal architecture, while retaining broadly similar global deformation patterns. Taxa: (A) Elephant, (B) *Plateosaurus*, (C) *Amargasaurus*, (D) *Diplodocus*, (E) *Giraffatitan*, (F) *Dreadnoughtus*, (G) *Australotitan*, (H) *Uberabatitan*, and (I) *Neuquensaurus*.. The color scale indicates displacement magnitude in mm, with white regions representing values that exceed the upper limit of the defined scale.

**Figure S4.** Distribution of nodal stress and displacement values in solid and native models. Kernel density estimates showing the distribution of Von Mises stress **(A)** and nodal displacement **(B)** for each taxon. Solid models are shown in blue and native models in orange. In most sauropodomorphs, native configurations alter the shape and spread of stress distributions relative to solid models, whereas displacement distributions remain concentrated near low values with taxon-specific differences in their upper ranges.

*References*

Campione, N. E. & Evans, D. C. (2012). A universal scaling relationship between body mass and proximal limb bone dimensions in quadrupedal terrestrial tetrapods. *BMC Biology*, 10, 60.

Campione, N. E., & Evans, D. C. (2020). The accuracy and precision of body mass estimation in non‐avian dinosaurs. *Biological Reviews*, *95*(6), 1759-1797.

Schaeffer, J. (2024). Osteological redescription of the holotype of Plateosaurus trossingensis (Dinosauria: Sauropodomorpha) from the Upper Triassic of SW Germany and its phylogenetic implications. *Journal of Systematic Palaeontology*, *22*(1), 2335387.
